# Feasibility of treatment planning with HyperArc stereotactic radiosurgery methods for ocular tumors

**DOI:** 10.1002/acm2.70399

**Published:** 2025-11-27

**Authors:** Chase Cochran, Shane McCarthy, William St. Clair, Damodar Pokhrel

**Affiliations:** ^1^ Medical Physics Graduate Program Department of Radiation Medicine University of Kentucky Lexington Kentucky USA; ^2^ Indiana University Health System Department of Radiation Oncology Indianapolis Indiana USA

**Keywords:** end‐to‐end test, HyperArc VMAT, Ocular/Intraocular Tumors, SRS

## Abstract

**Purpose/objectives:**

Currently, ocular disease is primarily managed with COMS plaque brachytherapy. Various stereotactic radiosurgery (SRS) platforms have also been employed, yet widespread access remains a challenge. Herein, we demonstrate the feasibility of using the HyperArc SRS system to provide an additional platform for the treatment of ocular malignancies.

**Materials/methods:**

Twenty previously treated COMS patients with ocular melanoma were selected for a retrospective HyperArc SRS planning study. The average gross tumor volume (GTV) derived from MRI was 1.19 ± 0.60 cc. Planning target volumes (PTV) were generated from a 2 mm expansion of the GTVs. HyperArc plans were created for a TrueBeam LINAC (6MV‐FFF) with the Encompass support system and used the AcurosXB algorithm for dose calculation. A dose of 25 Gy was prescribed to the PTVs and all plans were normalized such that PTVD95% = 25 Gy. All organs at risk (OARs) were spared adequately. Patient‐specific quality assurance (QA) and an independent Monte Carlo (MC) second check was performed for all SRS plans.

**Results:**

HyperArc plans demonstrated high conformality Paddick conformity index (PCI = 0.84 ± 0.04) with steep dose gradient index (GI = 3.29 ± 0.38). Compared to the HyTEC standard (12.1 Gy), it adequately spared the optic nerve(s) (*D*max = 7.18 ± 1.26 Gy, *p* < 0.001). Adequate PTV coverage (D99% = 23.80 ± 0.46 Gy) was achieved with a mean GTV dose of 27.54 ± 0.47 Gy. Maximum dose to critical OARs were controlled: brain (6.00 ± 1.50 Gy), optic nerve (7.18 ± 1.26 Gy), and ipsilateral lens (9.36 ± 5.67 Gy). Average beam‐on time was under 9 min. End‐to‐end QA results showed a 99.00 ± 0.59% pass rate (2%/2 mm γ‐criteria), with a MC check showing 97.81 ± 1.24% agreement with the AcurosXB algorithm (2%/2 mm).

**Conclusion:**

HyperArc SRS plans provided adequate target coverage, acceptable OARs sparing, and offer a fast, safe, and non‐invasive treatment option for ocular tumors. Clinical implementation of this novel methodology is ongoing at our institution.

## 1 INTRODUCTON

Stereotactic radiosurgery/radiotherapy (SRS/SRT) has emerged as a less invasive alternative to enucleation in the management of ocular malignancies, particularly uveal melanoma. Over the past several decades, a variety of SRS and brachytherapy techniques have been developed to deliver precise radiation to small, well‐defined ocular tumors while minimizing exposure to surrounding critical structures. These approaches include proton therapy, Gamma Knife SRS, robotic CyberKnife systems, and the COMS brachytherapy technique, each with unique advantages and limitations that depend on tumor characteristics, patient‐specific factors, and institutional capabilities.^1^, ^2^, ^3^, ^4^, ^5^, ^6^, ^7^, ^8^, ^9^, ^10^, ^11^, ^12^, ^13^ The major limitation of external beam approaches in radiotherapy for the treatment of ocular diseases lies in the motion management aspect. The inherent mobility of the eye being independent of the mobility of the body presents a unique and difficult challenge to navigate. Different external beam modalities have employed various motion management techniques with varying degrees of success and invasiveness including injecting retrobulbar anesthesia during simulation and treatment. In any case, motion management remains an ever‐present concern in any external beam radiation approach, and particularly for treating ocular tumors via SRS/SRT.

Proton therapy has demonstrated significant benefits in the treatment of various malignancies over the past few decades, largely due to its favorable dosimetric profile. Its application in the treatment of ocular tumors, especially uveal melanoma, has also shown encouraging results.^1^, ^2^ However, the limited global availability of proton therapy facilities, along with the high cost of operation, represents a significant barrier to its widespread clinical use.^3^ As a result, the clinical superiority of proton therapy over photon‐based modalities remains uncertain, with insufficient evidence to definitively establish its advantages.^4^

Gamma Knife SRS offers highly conformal dose distributions. It is particularly well‐suited for small, well‐defined intracranial lesions and is commonly used for central nervous system tumors. It has also been shown to provide effective treatment for ocular diseases.^5^, ^6^, ^7^ However, its use is limited by high costs, availability, and stringent radiation safety and security regulatory concerns. Additionally, the requirement of a head‐mounted frame‐based system increases the invasiveness of the treatment procedure and restricts the pool of eligible patients.

Robotic CyberKnife is a highly versatile SRS platform that offers significant advantages over traditional systems, particularly in terms of patient positioning and target mobility. Unlike fixed headframe systems such as the Gamma Knife, CyberKnife uses a robotic arm to deliver radiation to moving or irregularly shaped targets while simultaneously correcting setup errors. These features make CyberKnife well‐suited for SRS treatment procedures, including being used for treating ocular disease.^8^, ^9^, ^10^ However, despite its technical benefits, CyberKnife is limited by long treatment times and the need for advanced technical expertise. Additionally, the use of retrobulbar anesthesia for eye treatments increases its invasiveness.

The Collaborative Ocular Melanoma Study (COMS) brachytherapy procedure is considered the original gold standard for the treatment of many intraocular malignancies. It involves the surgical placement of a radioisotope‐containing eye plaque directly over the tumor. This enables the precise delivery of localized radiation with high precision. COMS plaques provide good local control rates comparable to more radical procedures, such as enucleation. However, the procedure is invasive, requires additional surgical resources, including access to an operating room, and is not suitable for all patients—particularly those with large or posteriorly located tumors, or those who are unable or unwilling to undergo surgery due to comorbidities or personal preferences. Radiation safety regulations around the medical use of radioactive materials prove to be another challenge for COMS plaques. Additionally, public safety concerns, due to the implantation of radioactive material, pose another challenge for patients and the facilities using this treatment approach. Therefore, availability of COMS brachytherapy centers is limited and the procedure is logistically complex, necessitating multidisciplinary coordination and long‐term postoperative follow‐up.^11^, ^12^, ^13^, ^14^ As a result, there is a clear need for an alternative LINAC‐based SRS approach that is non‐invasive, widely available, and capable of delivering highly conformal radiation to ocular tumors while minimizing the time and resource burden on patients and healthcare systems.

Developed by Varian Medical Systems (Palo Alto, CA), the HyperArc SRS module, offers a photon‐based SRS solution to intracranial disease that addresses several limitations of existing modalities as mentioned above. It utilizes an automated volumetric modulated arc therapy (VMAT) approach to deliver conformal dose distributions to small and complex intra‐axial targets.^15^, ^16^, ^17^, ^18^ Unlike proton therapy, it is available on stereotactic linear accelerators, which are more numerous, cost‐effective, and are readily available in many treatment centers, including community cancer centers. The HyperArc module employs a precise, frameless immobilization method via the Encompass support device and Q‐Fix mask (Q‐Fix, Avondale, PA) for patient set up. This frameless approach improves patient comfort compared to frame‐based methods such as those used in Gamma Knife SRS. Additionally, external beam radiotherapy approaches are not inherently limited by ocular tumor size and location, unlike COMS brachytherapy, which is typically reserved for smaller ocular lesions with no extraocular extension. Recent HyperArc studies have demonstrated its potential for treating skull‐based recurrent head and neck malignancies via SRT.^19^, ^20^

It is worth noting that other groups have explored VMAT‐based SRS/SRT for ocular tumors, but these efforts have often been institution‐specific approaches, lacking the standardization and reproducibility needed for broader clinical adoption.^21^, ^22^, ^23^ In contrast, this study presents a standardized SRS approach using the HyperArc platform for patient set up, treatment planning and delivery. By leveraging the infrastructure of HyperArc capable standard LINACs, this work seeks to establish the feasibility of HyperArc SRS as a reproducible and accessible option for treating ocular malignancies, particularly in settings where proton therapy, Gamma Knife, CyberKnife, or COMS plaques are not readily available. Additionally, this method could provide an alternative for scenarios where the invasiveness of the COMS brachytherapy procedure is not optimal for patients. In summary, while existing SRS methods and brachytherapy options have established clinical roles, they are not without limitations in terms of accessibility and invasiveness. This study aims to evaluate the technical aspect and clinical feasibility of HyperArc SRS for the treatment of ocular tumors through a retrospective planning and independent validation study.

## 2 MATERIALS AND METHODS

### 2.1 Patient selection and treatment planning

Patient selection presented a unique challenge for this study, as our institution currently utilizes COMS plaques for treating ocular malignancies. Consequently, no patients with both a HyperArc‐compatible CT scan and an ocular malignancy were available for inclusion. To overcome this, hybrid datasets were constructed from twenty anonymized patients that had HyperArc compatible CT scans (those acquired with the Encompass fixation device) and 20 additional anonymized MRI scans from past COMS patients. Scans were sourced from our institutional patient database after receiving institutional review board approval (IRB #92667). MRI scans were generated using an MPRAGE imaging sequence with gadolinium contrast and utilized a 1 mm slice thickness. The tumor geometry from the COMS patients’ MRIs was manually redrawn slice‐by‐slice to the existing HyperArc patient datasets to create the hybrid HyperArc patient datasets. These datasets incorporated realistic tumor contours while ensuring compatibility with the HyperArc treatment planning and delivery platform.

After generating the hybrid datasets, contouring was performed to delineate the critical organs, including the eyes, optic nerves, optic chiasm, lenses, lacrimal glands, brain, brainstem, and skin. The PTV was defined by a uniform 2 mm expansion of the GTV, consistent with margins used in other ocular SRS studies (Guberina et al.^21^ Tsui et al.^22^). A 2 mm margin was selected over 1 mm to provide a conservative scenario for this proof‐of‐concept study, acknowledging that larger volumes, generally increase proximity to OARs. Resulting GTV and PTV volumes were between 1.12 ± 0.61 cc and 2.79 ± 1.19 cc respectively. Patient‐specific tumor margins can be applied in an actual patient treatment including the use of asymmetrical margins.

Treatment planning was then carried out using the Eclipse Treatment Planning System (TPS) (Version 16.12, Varian Medical Systems, Palo Alto, CA). In order to remove planner experience as a confounding variable in plan quality, arc geometry, isocenter location, and collimator rotation was automatically selected by the HyperArc module with no intervention from the user. A primary objective of this study was to achieve adequate sparing of the optic pathway (optic nerves and optic chiasm), with particular emphasis on preserving the ipsilateral optic nerve. Adequate sparing of the optic nerve in this study was defined as a maximum dose of less than 12 Gy for a single fraction as defined by HyTEC guidelines.^24^ A dose of 25 Gy was prescribed to the PTV, and all plans were normalized to the point such that PTVD95% = 25 Gy. The SRS plans were designed for delivery on the TrueBeam platform using the standard Millenium 120 multileaf collimator (MLC) and a 6‐MV flattening filter‐free (FFF) beam. Final dose calculations were performed with the AcurosXB dose‐engine (v16.12) with a 1.25 mm dose calculation grid size and reported dose to medium.

### 2.2 Plan evaluation, quality assurance, and data analysis

Generated HyperArc SRS plans were subjected to evaluation using both quantitative metrics and qualitative assessments to ensure their clinical acceptability and dosimetric accuracy. Quantitatively, the SRS plans were evaluated using a variety of dosimetric indices, including the gradient index (GI), Heterogeneity index (HI) and Paddick conformity index (PCI).^25^, ^26^ Qualitatively, the plans were assessed by experienced radiation oncologists and medical physicists, who reviewed the dose distributions, and target coverage to ensure clinical acceptability. To ensure the accuracy and reliability of the treatment plans, patient‐specific quality assurance (PSQA) was performed using EPID‐based portal dosimetry.^27^, ^28^ The measured dose distributions from the EPID‐based PSQA were compared to the planned dose distributions using between 2%/2 mm and 3%/1 mm gamma criteria, both with a 10% threshold and a 90% passing criterion. Two gamma criteria were evaluated to represent our clinically used radiosurgery criteria of 2%/2 mm as well as a more stringent threshold of 3%/1 mm as recommended by various literature for SRS type treatments.^29^

Moreover, as an additional layer of independent verification, an in‐house Monte Carlo based second check was used. This Monte Carlo‐based^30^ dose calculation served as an independent verification of the AcurosXB dose algorithm within the Eclipse TPS. To reduce calculation times in our clinical practice, the dose calculation grid size is limited to 2 mm × 2 mm × 1 mm (slice thickness) to achieve a 3% statistical uncertainty. Due to this resolution limitation, only the 2%/2 mm threshold criteria were used for the MC second check process.

To assess plan metrics and gamma analysis results, all data was first tested for normality using statistical tests (e.g., Shapiro‐Wilk or Q–Q Plots). Based on the distribution of the data, parametric or non‐parametric statistical tests were applied accordingly. Specifically, for the results presented in this paper, the data was found to either be normally distributed or approximately normal, so all values of statistical significance were determined by conducting, a student's t‐test. Statistical significance was determined with a *p*‐value < 0.05. Moreover, mean, standard deviation, and range values for all plan evaluation metrics were reported.

## 3 RESULTS

### 3.1 Target coverage and conformity

**FIGURE 1:**
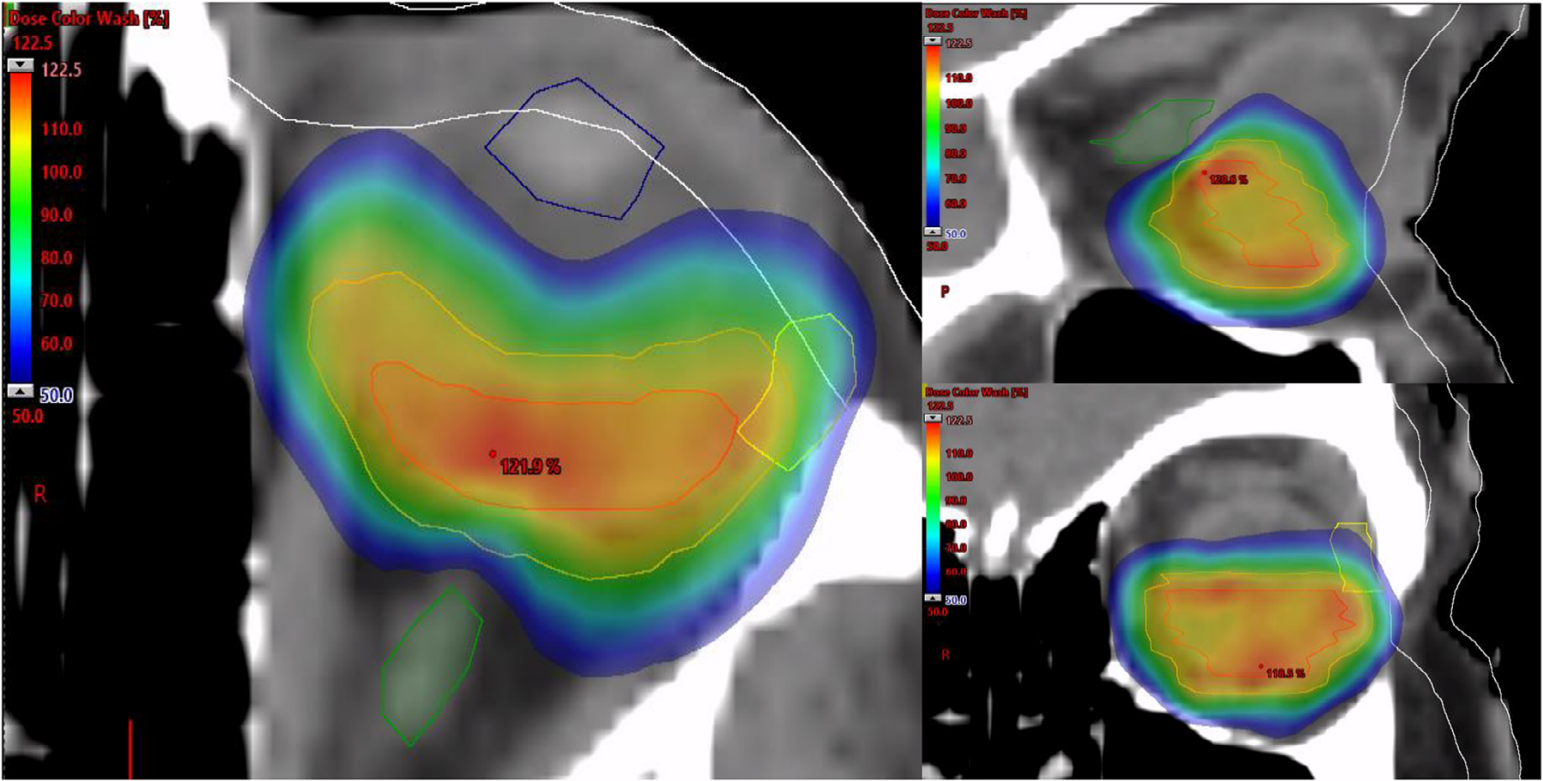
Demonstration of radiosurgical dose distribution for left ocular HyperArc stereotactic radiosurgery (SRS) plan (left: axial view, top right: sagittal view, bottom right: coronal view). In addition to the Planning target volumes (PTV) (Orange) and gross tumor volume (GTV) (Red) contours, the left optic nerve (green), lacrimal gland (yellow), lens (blue), and skin (White) are shown. Dose wash levels are shown including maximum dose (red), prescription dose of 25 Gy (green) and 50% of prescription dose (blue).

Table 1 shows the target quality metrics for the 20 generated HyperArc ocular SRS plans. Adequate tumor coverage (e.g., PTV mean dose of 27.54 Gy) of the target volume was observed while maintaining high conformity (RTOG CI = 1.08, PCI = 0.84). Additionally, GTVs received sufficient dose (D99% = 25.48 Gy). The plans also featured sharp dose gradients (average GI = 3.29), reducing the expansion of the 50% isodose line, which coincides with the optic nerve limit. PTV ICRU83 homogeneity index (HI = (D_2_‐D_98_)/D_presc_) values which measure the homogeneity of the dose distribution within the target, were measured to be 0.24 ± 0.05 (0.11 – 0.33) corresponding to global maximums of 31.25 ± 1.20 (28.03 – 32.93) Gy. Figure 1 shows a HyperArc SRS plan for one of the patients with a left‐sided ocular tumor (PTV = 3.98 cc). The plan consisted of three left‐sided partial arcs delivering a prescription dose of 25 Gy in a single fraction. Highly conformal radiosurgical dose distributions (PCI = 0.88) are shown, in addition to, adequate sparing of adjacent critical organs, specifically the left optic nerve (Dmax = 7.12 Gy).

**TABLE 1:** Plan quality metrics for tumor dose, PTV coverage and conformity for all 20 ocular SRS plans evaluated via HyperArc geometry. Mean ± std. dev. (range) is reported.

Parameters	Global	
*D*_max_ (Gy)	31.25 ± 1.20 (28.03 – 32.93)	

	PTV	GTV
*D*_99%_ (Gy)	23.80 ± 0.46 (22.48 – 24.34)	25.84 ± 0.43 (24.98 – 26.84)
*D*_mean_ (Gy)	27.54 ± 0.47 (26.30 – 28.47)	28.19 ± 0.63 (26.65 – 29.30)
RTOG CI	1.08 ± 0.06 (1.00 – 1.21)	N/A
PCI	0.84 ± 0.04 (0.75 – 0.90)	N/A
GI	3.29 ± 0.38 (2.69 – 4.08)	N/A
HI	0.24 ± 0.05 (0.11 – 0.33)	N/A

Note: Planning target volumes; PTV; stereotactic radiosurgery; STV; Heterogeneity index; HI; Paddick conformity index; PCI; gradient index; GI; stereotactic radiosurgery; SRS.

### 3.2 Dose to adjacent OARs

Table 2 shows dose to various adjacent OARs from the generated HyperArc SRS plans. The maximum dose to OARs were found to be within satisfactory limits with the optic nerve dose being well below the 12 Gy limit. OARs not located within the immediate vicinity of the treated eye (contralateral eye, chiasm, brain) received relatively low amounts of dose for SRS treatment. The greatest maximum dose (D0.03cc) received by the brain in a worst‐case scenario was 8.9 Gy. Which is promising in terms of the standard of evaluating radio necrosis risk based on V14Gy.^31^ Maximum dose to OARs varied widely as a function of tumor size and location. In some cases, such as the PTV overlapping the lacrimal gland for lateral tumors, sparing of the lacrimal gland may not be feasible. For most cases, sparing of the skin (Dmax < 26 Gy) was possible, however for more anteriorly located tumors, staying under this limit with the 25 Gy prescription dose was not possible. In the end, coverage of the PTV was prioritized. In cases such as these, different fractionation schedules (e.g., 3 Fx, or 5 Fx) may be required to reduce adverse reactions.

**TABLE 2:** Data showing summary of maximum dose to OARs of HyperArc SRS plans for all 20 ocular SRS plans. *P*‐values used to compare optic pathway maximum dose limit (< 12 Gy) set by HyTEC guidelines.^24^ Mean ± std. dev. (range) is reported. *P*‐values < 0.05 are in bold.

Ipsilateral Optic Nerve (Gy)	7.18 ± 1.26 (4.51 – 9.88)	**< 0.001**
Contralateral Optic Nerve (Gy)	1.10 ± 0.59 (0.38 – 2.23)	**< 0.001**
Ipsilateral Lens (Gy)	9.36 ± 5.67 (3.97 – 27.28)	N/A
Ipsilateral Lacrimal Gland (Gy)	15.52 ± 9.47 (3.24 – 31.40)	N/A
Optic Chiasm (Gy)	0.89 ± 0.57 (0.29 – 2.66)	**< 0.001**
Brainstem (Gy)	0.99 ± 0.43 (0.47 – 1.71)	N/A
Brain (Gy)	6.00 ± 1.50 (3.55 – 8.9)	N/A
Skin‐PTV (Gy)	22.88 ± 5.38 (6.91 – 27.67)	N/A

Note: stereotactic radiosurgery; SRS.

### 3.3 Treatment delivery efficiency and accuracy

Table 3 shows the clinical validation results of the HyperArc SRS plans, which included EPID‐based portal dosimetry PSQA and an independent TPS check using an in‐house Monte Carlo (MC) dose calculation. EPID‐based PSQA results were satisfactory for clinical use. Using a 90% passing criterion, all SRS plans passed 2%/2 mm gamma analysis, 3 of the 20 plans failed using 3%/1 mm criteria. Correlation tests were conducted between the 3%/1 mm PSQA pass rates and various plan metrics (target location, target volume, beam on times, etc.) to determine if any relationship could be derived to address why certain plans failed. Modulation factor (MF) which is calculated as the ratio of the MUs required by the plan to the prescription dose in cGy, was determined to have a negative correlation (−0.53) with PSQA pass rates for these SRS plans. This indicates that possibly reducing plan modulation either via re‐optimization with more aggressive aperture shape controller settings or by setting lower MU objectives, may potentially improve the PSQA pass rates. MC‐based second check results were, on average, lower and more varied compared to the EPID‐based PSQA results. Still, for the 2%/2 mm gamma passing criteria, all plans passed. This general overall decrease compared to the EPID‐based QA was attributed to the simplified radiation transport method and MLC modeling uncertainty in the MC code.^31^ This leads to compounded errors in highly modulated, small field plans like the ones reported here.

**TABLE 3:** Summary of treatment delivery parameters and PSQA parameters for all 20 HyperArc VMAT ocular SRS patients. Mean ± std. dev. (range) is reported.

Monitor Units (MU)	11513.1 ± 1788.6 (8165.8 – 13455.8)	
Modulation Factor (MF)	4.61 ± 0.72 (3.27 – 5.38)	
Beam on Time (mins)	8.22 ± 1.28 (5.83 – 9.61)	
Gamma Passing Criteria	**2%/2** **mm**	**3%/1** **mm**
MC Second Check (% Agreement)	97.81 ± 1.24 (94.30 – 99.60)	N/A
EPID‐based PSQA Pass Rate (%)	99.00 ± 0.59 (98.00 – 100.00)	94.25 ± 2.51 (82.00 – 98.30)

Note: stereotactic radiosurgery; SRS; patient‐specific quality assurance; PSQA; volumetric modulated arc therapy; VMAT.

In terms of deliverability, HyperArc‐generated ocular SRS plans were shown to be highly modulated. Across all plans, MF = 4.61 ± 0.72 (3.27–5.38). Utilizing a 6 MV‐FFF beam (1400MU/min), beam on time on average was 8.22 mins with a minimum and maximum beam on time between 5.83 and 9.61 mins, respectively. Minimizing beam on times is paramount to patient comfort and reducing intra‐fractional motion.

**FIGURE 2:**
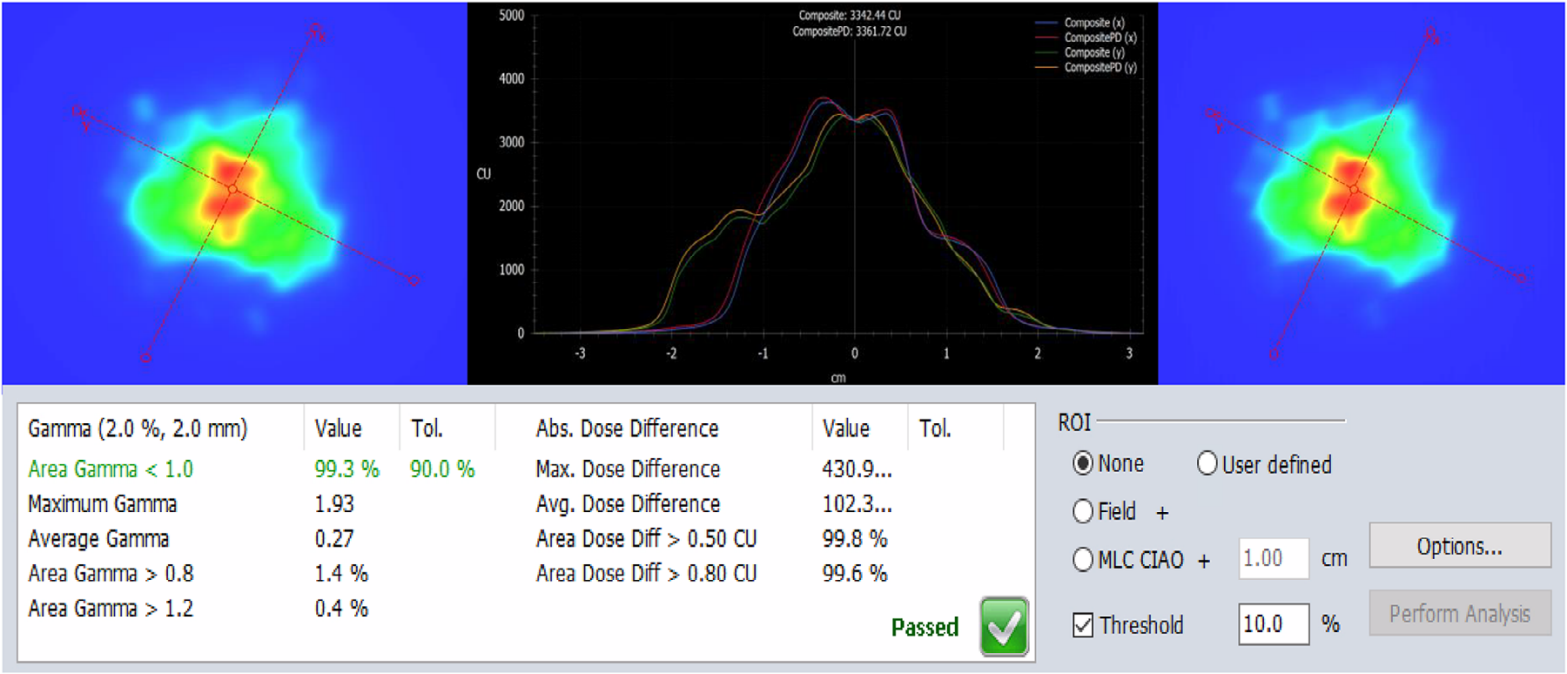
Demonstration of the EPID‐based PSQA results for an example HyperArc brain stereotactic radiosurgery (SRS) patient, calculated composite PD fluence map (left) vs delivered fluence map (right) and superimposed both the x‐ and y‐ dose profiles (middle) are shown–indicating a clinically acceptable gamma passing rate of 99.3% using a 2%/2 mm threshold.

Figure 2 represents our clinical results of EPID‐based PSQA for an example HyperArc SRS patient as shown in Figure 1. Although the AAPM reports suggest using 3%/2 mm gamma criteria,^27^, ^28^ due to the small pixel size of the aS1200 EPID detector (0.34 mm), in our clinic, we routinely use a tighter 2%/2 mm clinical gamma passing criteria for these small‐field HyperArc SRS plans. 3%/1 mm values are also reported (see Table 2). The results demonstrate high QA passing rates despite the high‐modulation of the field and small size of the tumors.

### 3.4 Comparison to other modalities

Various other external‐beam radiation‐based approaches have been used for the treatment of ocular diseases. Comparison to existing, proven methodologies is required to gauge the performance of the HyperArc platform. A wide array of prescription doses and fractionation schemes are used and therefore, direct comparisons are difficult. Below, various comparisons are made to different treatment methods and in order to compare between fractionation schemes, dose values are reported as both raw values in Gy and percentage of total prescription dose.

#### 3.4.1 Non‐coplanar VMAT SRS

Guberina et al.^21^ utilized a non‐coplanar VMAT based approach in conjunction with retrobulbar anesthesia during treatment. Number of arcs averaged 5 (4‐12) and special care was taken to minimize brain entrance and exit dose. VMAT results compared to our HyperArc SRS below (Table 4).

**TABLE 4:** Plan metric comparison to Guberina et al. Values reported as average raw data (Gy) / percentage of total prescription values (where applicable).

Metric	Guberina et al. (22 Gy in 1 Fx)	This study (25 Gy in 1 Fx)
PTV *D*_99.5%_	22.12 Gy / 101.00%	23.28 Gy / 93.12%
PTV *D*_mean_	26.67 Gy / 121.23%	27.54 Gy / 110.16%
CI	1.20	1.08
GI	2.60	3.29
*V*_50_ [cc] / PTV [cc]	3.12	3.59
Brain *D*_mean_	0.14 Gy / 0.63%	0.36 Gy / 1.44%
Brain *D*_1cc_	1.7 Gy / 6.80%	4.4 Gy / 17.6%
Optic Nerve *D*_max_	7.77 Gy / 31.08%	7.18 Gy / 28.72%

Conformity Index; CI; Gradient Index; GI; Planning target volumes; PTV.

#### 3.4.2 DCA‐IMRT Ocular SRT

Another study by Ciernik et al.^32^ compared the performance of DCA‐IMRT against a traditional VMAT based approach for treating ocular tumors and found the DCA‐IMRT method to be superior. Results are from the DCA‐IMRT method and are compared against the HyperArc SRS method (Table 5).

**TABLE 5:** Plan metric comparison to Ciernik et al. raw data. Values reported as percentage of total prescription (where applicable) due to the different fractionation schemes.

Metric	Ciernik et al. (50 Gy in 5‐Fx)	This study (25 Gy in 1 Fx)
PTV V95%	98.30%	99.14%
PTV *D*_max_ ^*^	<107.00%	124.92%
CI	1.24	1.08
HI	0.05	0.24
Optic Nerve *D*_max_	7.77 Gy / 31.08%	7.18 Gy / 28.72%
Lens *D*_max_ ^*^	33.17 Gy / 66.34%	9.36 Gy / 37.44%
Lacrymal Gland *D*_max_	24.60 Gy / 49.20%	10.03 Gy / 40.12%

* D_max_ not reported directly but V107% found to be 0 across all SRS plans.

Note: Conformity Index; CI; Gradient Index; GI; Planning target volumes; PTV.

## 4 DISCUSSION

### 4.1 Overview

In this retrospective planning and validation study, we evaluated the plan quality, treatment delivery efficiency, and accuracy of ocular/intraocular SRS treatments using the automated HyperArc module with 6MV‐FFF beams (1400 MU/min) on the TrueBeam LINAC. Our results demonstrate that HyperArc SRS is a feasible and highly efficient treatment modality for treating extracranial ocular/intraocular tumors. HyperArc‐generated plans exhibited excellent dose conformity, steep dose gradients, and demonstrated the ability to efficiently spare adjacent critical OARs including the optic pathway. For tumors that are closer to the optic nerve or where OAR dose limits are difficult to meet, fractionated SRS (3‐5 fractions) can be delivered via this method.

### 4.2 Comparison to other methods

In comparison with other treatment modalities, HyperArc performed well. Since the results in this study are based on a single fraction, the most direct comparison can be made with the study by Guberina et al.^21^ which used a similar dose and single‐fraction approach. When evaluated as a function of prescription dose, the PTV minimums (D99.5%) reported by Guberina et al. were 101%, compared to 93.12% for HyperArc. The mean doses to PTVs were 121% for Guberina et al. and 110% for HyperArc. CI favored HyperArc (1.08 vs. 1.20), while the GI and V50/PTV both favored the manual approach described by Guberina et al. Brain dose metrics also favored the manual approach, with lower mean and maximum doses reported by Guberina et al. In contrast, the maximum dose to the optic nerve favored HyperArc, despite the higher prescription dose. However, it should be noted that optic nerve dose varies significantly depending on the tumor's proximity to the nerve; therefore, direct comparisons between the two methods should be interpreted with caution. When compared to SRT by Ciernik et al.^32^ target coverage metrics favored the HyperArc approach, showing improved coverage and higher Dmean values. Ciernik et al. reported very low Dmax values (<107%), indicating more homogeneous dose distributions, whereas HyperArc produced higher maximum doses (124%). With respect to OARs, both absolute dose values and doses expressed as a percentage of prescription favored HyperArc. However, since the current results are based on a single fraction, and Ciernik used fractionated schemes, the apparent advantage in OAR sparing cannot be directly interpreted. Overall, comparisons of HyperArc to other techniques showed mixed results: HyperArc achieved superior plan quality in some metrics and inferior performance in others. Importantly, all reported values in this study remained within clinical guidelines, demonstrating that HyperArc can perform comparably to other established methods while offering the added benefit of partial planning and delivery automation.

### 4.3 Current limitations and ongoing work

While this study demonstrates promising dosimetric and quality assurance results using HyperArc. Some limitations should be acknowledged. First, the retrospective nature of this study limits the ability to assess any patient outcome data of this modality. Additionally, while the sample size of 20 ocular SRS cases is sufficient for a proof‐of‐concept feasibility study, the results may not be generalizable to other institutions or patient populations with their available technology/cohort. The SRS plans were generated using a TrueBeam LINAC with the standard Millenium 120 MLCs (5 mm width). High‐definition 2.5 mm MLCs are expected to further enhance the dosimetric quality of the plans. Nevertheless, the results obtained with the standard MLC system are promising, suggesting that such machines are still viable for this treatment modality. The patient selection process was also challenging, and the use of hybrid patient datasets that required manual recreation may have introduced some bias that we would like to mention. Special care was taken to ensure accuracy between the targets on the MRI images and their recreation on the CT images. Manual contouring inevitably introduces variability in plan contours, as it depends on the SRS planner's experience and judgment. Furthermore, this study did not address the issue of ocular motion monitoring during treatment delivery yet, which presents a significant challenge on eye tumor patients due to the eye's natural mobility. The introduction of eye immobilization devices, commonly head mounted solutions, may prove to be incompatible with the HyperArc treatment geometry, limiting its utility in the treatment of ocular diseases. Our ongoing research work is focusing on addressing these current limitations. Clinical implementation of this SRS treatment method is currently underway at our institution, which will enable the collection of more robust patient datasets and the assessment of clinical outcomes in the future. Development of a motion management method for ocular treatments is also planned. Additionally, the integration of automated RapidPlan modeling is being explored to enhance the efficiency of the ocular SRS treatment planning process and reduce inter‐planner variability.

## 5 CONCLUSION

For ocular/intraocular tumors, HyperArc SRS offers a highly conformal dose distribution with a rapid dose fall‐off and excellent sparing of adjacent critical structures. It will enable precise and accurate treatments of ocular tumors, making it an attractive treatment option for patients who cannot tolerate, deny or are not eligible for COMS brachytherapy or other ocular SRS procedures. Additionally, the widespread availability of LINAC‐based SRS, including the automated HyperArc module, increases access to care for this patient cohort. Due to its exceptional plan quality and treatment delivery efficiency, the clinical implementation of stereotactic treatments for ocular/intraocular tumors using the HyperArc module is currently underway at our institution. While this study demonstrates promising dosimetry and quality assurance results, further investigation is warranted to evaluate long‐term clinical outcomes and to derive proper motion management procedures that are compatible with the HyperArc platform.

## AUTHOR CONTRIBUTIONS

Damodar Pokhrel and Chase Cochran conceived the project. Damodar Pokhrel generated all HyperArc plans. Chase Cochran, Shane McCarthy and Damodar Pokhrel collected and analyzed data and completed the end‐to‐end and validation tests. William St. Clair and Damodar Pokhrel provided their clinical expertise and supervision of the research. Chase Cochran and Damodar Pokhrel drafted the preliminary manuscript and all co‐authors revised and approved the final manuscript for submission.

## CONFLICT OF INTEREST STATEMENT

Authors reported no conflict of interest.

## Data Availability

Research data are not shared.
